# THE RELATIONSHIP BETWEEN MARITAL STATUS, COHORT, AND DEPRESSION SYMPTOMS IN TAIWAN

**DOI:** 10.1093/geroni/igz038.1140

**Published:** 2019-11-08

**Authors:** Fang-Yi Huang, Min Li

**Affiliations:** 1 Trinity Washington University, College Park, Maryland, United States; 2 University of Florida, GNV, Florida, United States

## Abstract

Objectives: The relationship between marital status and depression symptoms is well documented. However, how the negative economic shock affect relationship differ by gender and cohort is still indecisive. The dataset “2011 wave of the Taiwan Longitudinal Study in Aging” and logistic regression models were used in the study. The results: Marital status is related to depression symptoms, but it differs by gendered cohort. With considering financial shock, there is no difference of depressive symptom between divorced and married female. The divorced and widowed have 4.81 and 2.47 times higher of getting depression symptom than the married for baby boom female. Being divorced is 3.67 times higher of getting depressive symptoms than being married for baby boom male. For WWII female, the widows are 1.78 times higher to have depressive symptoms than the married. being divorced, widowers, and single are 3.32, 2.21 and 2.90 times higher of getting depressive symptoms than being married for WWII male. Being divorced is 3.67 times higher of getting depressive symptoms than being married for baby boom male. In conclusions, people with unstable marital statuses are more depressed than the married. In particular, the effect of unstable marital statuses on depression could be account for by financial decline for women but not men. Given the policy emphasis on those with unstable marital status and economic decline, divorce female and single baby boom female may represent particular groups in whom interventions designed to financially support.

